# Investigating LATS1 and NF-κB as Predictors of Radiotherapy Response in Cervical Cancer

**DOI:** 10.3390/cimb47050365

**Published:** 2025-05-16

**Authors:** Andi Darma Putra, Andrijono, Hariyono Winarto, Ani Retno Prijanti, Lisnawati, Trevino Aristarkus Pakasi, Supriadi Gandamihardja, Jourdan Wirasugianto, Amelia, Lasmini Syariatin

**Affiliations:** 1 Doctoral Program in Medical Sciences, Faculty of Medicine, Universitas Indonesia, Central Jakarta 10430, Indonesia; 2 Division of Gynecology-Oncology, Department of Obstetrics and Gynecology, Faculty of Medicine, Universitas Indonesia, Cipto Mangunkusumo Hospital, Central Jakarta 10430, Indonesia; andrijono@gmail.com (A.); hariyono.winarto@ui.ac.id (H.W.); 3 Dopamine Science Institute, Depok 16431, Indonesia; lasminisyariatin@mail.ugm.ac.id; 4 Department of Biochemistry and Molecular Biology, Faculty of Medicine, Universitas Indonesia, Cipto Mangunkusumo Hospital, Central Jakarta 10430, Indonesia; ani.retno@ui.ac.id; 5 Department of Pathological Anatomy, Faculty of Medicine, Universitas Indonesia, Cipto Mangunkusumo Hospital, Central Jakarta 10430, Indonesia; lisnawatidr@gmail.com; 6 Department of Community Medicine, Faculty of Medicine, Universitas Indonesia, Cipto Mangunkusumo Hospital, Central Jakarta 10430, Indonesia; trevino.ap@ui.ac.id; 7 Division of Gynecology-Oncology, Department of Obstetrics and Gynecology, Faculty of Medicine, Universitas Padjadjaran, Hasan Sadikin Hospital, Bandung 40161, Indonesia; supriadigand@yahoo.co.id; 8 Assistant Division of Gynecology-Oncology, Department of Obstetrics and Gynecology, Cipto Mangunkusumo Hospital, Central Jakarta 10430, Indonesia; jourdanwirasugianto@gmail.com (J.W.); amelsumber@gmail.com (A.)

**Keywords:** biomarker, cervical cancer, LATS1, NF-κB, radioresistance, radiotherapy

## Abstract

Cervical cancer is the fourth most prevalent cancer among women globally. Protein concentrations of Large Tumor Suppressor Kinase-1 (LATS1) and Nuclear Factor Kappa-B (NF-κB) have been identified as prospective biomarkers of radioresistance in cervical cancer. This preliminary study aimed to investigate the effectiveness of LATS1 and NF-κB levels as a biomarker for radioresistance and evaluate their response to radiation in cervical cancer patients. A comprehensive cross-sectional study was conducted involving 114 subjects diagnosed with advanced stages cervical cancer (stage IIIB and IVA) who underwent definitive radiotherapy. The concentrations of LATS1 and NF-κB were measured using ELISA from biopsy samples taken prior to the initiation of radiotherapy. This study’s finding included 114 subjects, with a median age of 53 years. A total of 85 (74.5%) subjects had stage IIIB, while 29 (25.4%) subjects had stage IVA. The cut-offs for LATS1 and NF-κB were 0.02765 ng/mg and 192.42 pg/mg, respectively. Subjects with a higher expression of LATS1 were found to be unresponsive to radiation therapy (*p* ≤ 0.001; AUC = 32.7%), and subjects with a lower expression of NF-κB were found to be unresponsive to radiation therapy (*p* = 0.009; AUC = 61%). This study suggests that elevated LATS1 expression may inversely predict radioresistance, while NF-κB expression shows a weak correlation with resistance to radiation therapy.

## 1. Introduction

Cervical cancer is the fourth most prevalent cancer in women globally. In 2022, there were approximately 662,301 new cases diagnosed, with 348,874 deaths resulting from the disease [1]. Projections from The American Cancer Society for 2025 estimate approximately 13,360 new cases of invasive cervical cancer in the United States, with approximately 4320 deaths expected from the disease [2]. In Indonesia, cervical cancer presents a significant health challenge, particularly among women aged 15–44 years, where it ranked as the second most common cancer [3,4,5].

A considerable proportion of cervical cancer cases are detected at advanced local stages, and radiation therapy, often in combination with cisplatin chemotherapy, is the primary treatment option [6,7]. However, resistance to radiotherapy poses a major challenge for its effective treatment [8]. Investigating biomarkers, genetic pathways, cellular changes in irradiated cells, and the tumor microenvironment is essential for understanding radioresistance [9,10].

Recent studies have explored the biological function of Large Tumor Suppressor Kinase-1 (LATS1) in tumors, focusing on its role in regulating the cell cycle, differentiation, and motility through the Hippo signaling pathway [11,12]. Although dysregulation of LATS1 has been linked to cancer development and progression, its clinical value as a validated biomarker remains under investigation [13,14,15]. As a serine/threonine kinase, LATS1 is essential in the mitotic apparatus, undergoing phosphorylation in cell-cycle-dependent pathways, suggesting its potential as a negative regulator of CDC2/cyclin-A activity [16,17]. Inside the Hippo pathway, LATS1 plays a crucial role in controlling adult cell growth and affects cell proliferation, differentiation, and migration during organ development [18].

Apoptosis plays a crucial role in the elimination of cancer cells during radiation therapy. The NF-κB activation pathway plays a key role in the disruption of apoptosis [19]. NF-κB, a group of transcription factors, promotes cell survival and proliferation while also regulating the immune system and inflammatory responses. In normal cells, NF-κB activation suppresses cell death by inducing genes that regulate mitochondrial and death receptor pathways [20]. NF-κB also promotes the production of inhibitors of apoptosis (IAPs) and different members of the anti-apoptotic *bcl-2* family. In addition, NF-κB can inhibit the ability to hinder the transcriptional activity of p53 by upregulating anti-apoptotic genes and downregulating p53, thereby inhibiting p53-induced apoptosis [19].

The transcription factor NF-κB is believed to contribute to cellular resistance to apoptosis, potentially affecting the cellular response to radiation. Radiation-induced cell death occurs due to both direct DNA damage and free radical formation [21]. The ability of cancer cells to maintain their levels of reactive oxygen species (ROS) levels is a critical mechanism in the process of radioresistance, involving antioxidant activity or free radical scavenging. Following radiation exposure, increased intracellular ROS levels can activate NF-κB. Upon activation, NF-κB triggers the activation of different genes and kinases, such as *bcl-2*, bcl-xL, XIAP, survivin, and Akt, which regulate resistance and contribute to radioresistance [22].

In advanced cervical cancer, LATS1 and NF-κB play similar roles in contributing to radioresistance. However, it is still uncertain whether these factors can accurately predict the response to chemoradiotherapy in locally advanced-stage cervical cancer. Although dysregulation of LATS1 and NF-κB has been associated with radioresistance in cervical cancer cells, research in this area is limited [23]. Evaluating the levels of LATS1 and NF-κB in patients with advanced cervical cancer receiving radiotherapy could provide valuable insights as prognostic markers. Nevertheless, there are challenges in fully understanding the roles of these biomarkers in radiation therapy, particularly in advanced-stage cervical cancer.

## 2. Materials and Methods

### 2.1. Study Design and Ethical Clearance

This comprehensive cross-sectional study was conducted at Cipto Mangunkusumo Hospital, Central Jakarta, Indonesia, from 1 July 2017 until 30 June 2023, focusing on patients diagnosed with stage IIIB and IVA cervical cancer who underwent definitive radiotherapy based on the revised 2018 International Federation of Gynaecology and Obstetrics (FIGO) staging. Medical records for subjects were maintained in accordance with relevant ethical standards, and informed consent was obtained from all subjects prior to the study. This study was approved on 15 March 2021 by the Ethics Committee of the Faculty of Medicine, University of Indonesia, Cipto Mangunkusumo Hospital (ethical clearance certificate no: KET-245/UN2.F1/ETIK/PPM.00.02/2021).

### 2.2. Patient and Radiation Response Criteria

The assessment criteria for determining the response of cervical cancer patients undergoing radiation therapy adhered to the RECIST 1.1 guidelines [24]. The criteria for patient inclusion in this study included individuals diagnosed with stage IIIB and IVA cervical cancer who were scheduled to receive definitive radiotherapy in conjunction with cisplatin. Additionally, it required the availability of paraffin block preparations and cervical biopsies dissolved in DNA solution. Histopathological examination revealed squamous cell carcinoma, adenocarcinoma, and other specified types. Furthermore, patients had to not exhibit metastases, metabolic disorders, infections, or any other critical health conditions following thorough laboratory assessments, chest imaging, and ultrasound evaluations. The criteria for achieving a complete response following radiation treatment stipulated that all target lesions had to be reduced by at least 10 mm. A partial response was indicated when there was a reduction of at least 30%. Partial response (PD) criteria were defined when there was a minimum 20% increase in the diameter of the target lesion. If there was no reduction in PR or PD, the condition was classified as stable disease (SD).

### 2.3. Reagents

LATS1 levels were measured using a sandwich-enzyme-linked immunosorbent assay (ELISA) technique with a commercial kit from MyBioSource Inc. (cat. no. #MBS9329510, MyBiosource Inc., San Diego, CA, USA) (Mybiosource.com, 2020). NF-κB levels were assessed using a sandwich enzyme immunoassay technique with a commercial kit (product no. #SEB824Hu, USCN Inc., Wuhan, China). The procedures involved the use of horseradish peroxidase (HRP) conjugate reagent and phosphate-buffered saline (PBS).

### 2.4. Sample Preparation

LATS1 and NF-κB levels were measured using a sandwich-enzyme-linked immunosorbent assay (ELISA). Biopsy tissue samples were initially processed by removing excess blood to minimize contamination, followed by homogenization to create a uniform mixture. The homogenized tissue was then mixed with phosphate-buffered saline (PBS) at a ratio of 100 µL of PBS per 10 mg of tissue, ensuring stable pH and ionic conditions. The mixture was subsequently centrifuged at 1500× *g* for 15 min to separate the soluble proteins from the cellular debris. The resulting supernatant was carefully collected and stored in aliquots at −20 °C to maintain protein integrity until further analysis.

### 2.5. ELISA Assay for LATS1 Level

The ELISA was performed according to the manufacturer’s instructions. The reagents were equilibrated to room temperature (18–25 °C) for 30 min before the assay began. For the assay setup, 50 µL of standard solution was added to the standard wells, 50 µL of diluent was added to the control wells, and 50 µL of the sample was added to the sample wells. Subsequently, 100 µL of horseradish peroxidase (HRP) conjugate reagent was added to each well. The plate was then sealed with an adhesive and incubated at 37 °C for 60 min.

Following the incubation, the wells were thoroughly washed up to four times to remove any unbound substances. Next, 50 µL each of chromogen solutions A and B were added to each well, and the plate was incubated for 15 min at 37 °C in the dark to allow for color development. To halt the reaction, 50 µL of stop solution was added to each well, resulting in a color change from blue to yellow. If the color was uneven, the plate was gently tapped to mix the contents. The optical density (OD) was measured at 450 nm using a Microelisa Strip Plate Reader within 15 min of adding the stop solution. The detection range for LATS1 was between 0.25 and 8.0 ng/mL, as specified by the kit.

### 2.6. ELISA Assay for NF-κB Level

The levels of NF-κB were quantified using a sandwich-enzyme-linked immunosorbent assay with a commercial kit. This process began with a microtiter plate pre-coated with monoclonal antibodies specific to NF-κB. After preparing reagents, samples, and standards, 100 µL of each standard or sample was added to the wells and incubated at 37 °C for 2 h to allow for NF-κB binding. Following this, 100 µL of a biotinylated detection antibody (detection reagent A) was added and incubated for 1 h at 37 °C. The wells were then aspirated and washed three times to remove unbound materials.

Next, 100 µL of an avidin-HRP conjugate (detection reagent B) was added, binding to the biotinylated antibodies, and the plate was incubated for 30 min at 37 °C. After five additional washes to eliminate excess conjugate, 90 µL of TMB substrate was added, which reacted with HRP to produce a color change. This reaction was allowed to develop for 15–25 min at 37 °C, followed by the addition of 50 µL of stop solution. The optical density was then measured at 450 nm using a spectrophotometer. Finally, the concentration of NF-κB was determined by comparing the OD readings to a standard curve, enabling accurate quantification of the NF-κB levels in the samples.

### 2.7. Statistical Analysis

Data were analyzed using an independent *t*-test to compare means between two groups for normally distributed data, while the Mann–Whitney U test was applied for non-normally distributed data. Statistical significance was set at *p* < 0.05. Receiver Operating Characteristic (ROC) analysis was used to determine the cut-off values. The results of the multivariate analysis were evaluated in terms of pre-test probability, post-test probability, sensitivity, specificity, positive predictive value (PPV), negative predictive value (NPV), likelihood ratio (LR), pre-test odds ratio, post-test odds ratio, and confidence intervals. Sample size calculation was performed using a rule of thumb approach, which suggested that a minimum of 30 patients per group were required to achieve sufficient statistical power for the analyses. The research data were processed electronically using the SPSS Statistics version 23.0 software (IBM Corporation, Armonk, NY, USA).

## 3. Results

### 3.1. Clinical Data

This study included participants diagnosed with advanced cervical cancer at stages IIIB and IVA, with a focus on analyzing the levels of LATS1 and NF-κB and their association with radiation therapy outcomes, as assessed according to the RECIST 1.1 criteria [24]. A total of 114 individuals, with a median age of 53 years, were enrolled in the study. Among these, 87 participants (76.3%) were married, and 60 participants (52.6%) had a parity greater than 2 (Table 1).

The distribution of cancer stages revealed that 85 participants (74.5%) were diagnosed with stage IIIB cervical cancer, while 29 participants (25.4%) were diagnosed with stage IVA. Regarding radiation response, 30.7% of the participants achieved a complete response, 59.6% experienced disease progression, and 9.6% exhibited a partial response.

### 3.2. Correlation Between LATS1 and NF-κB Levels of Expression and Radiation Therapy Response

LATS1 and NF-κB expression levels were analyzed in 114 samples. The findings revealed a significant increase in LATS1 expression and a decrease in NF-κB expression among subjects who did not respond to radiation treatment. The Mann–Whitney test further confirmed this observation, showing that the group exhibiting radioresistance demonstrated an elevated median and interquartile range for LATS1 while showing a diminished median and interquartile range for NF-κB compared to the radiosensitive group. Additionally, a significant difference in LATS1 and NF-κB levels was observed between the radiation response groups, with *p*-values of 0.001 and 0.045, respectively (Table 2). These results suggest a strong correlation between elevated LATS1 expression and reduced NF-κB expression and radioresistance in cancer patients.

**Figure 1:**
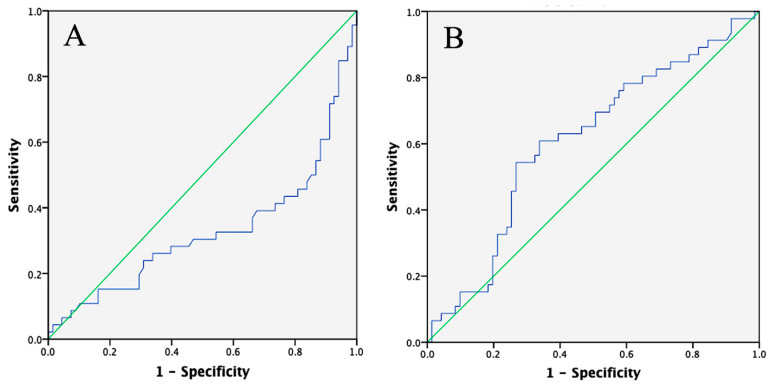
ROC curve of LATS1 and NF-κB. (**A**) ROC curve of LATS1 shows an AUC of 32.7%, (**B**) ROC curve for NF-κB shows an AUC of 61%.

The cut-off values established for LATS1 and NF-κB were 0.02765 ng/mg and 192.42 pg/mg, respectively. ROC analysis indicated that the area under the curve (AUC) for LATS1 was 32.7%, suggesting that LATS1 has an inverse predictive value as a candidate biomarker for radioresistance in patients with advanced cervical cancer. In contrast, NF-κB had an AUC of 61%, reflecting weak predictive power (Figure 1). The proportion of patients with LATS1 levels above the cut-off was significantly higher in the radioresistant group than in the radiosensitive group (*p* < 0.001). There was also a significant difference in NF-κB levels below the cut-off between the two groups (*p* = 0.009), with higher levels observed in the radiosensitive group.

In patients whose LATS1 examination results exceeded the established cut-off point, the probability of exhibiting radioresistance was found to be 71.5%. This finding suggests a significant likelihood of radioresistance in these individuals. On the other hand, patients whose NF-κB examination results fell below the cut-off had a 69% probability of being radioresistant (Table 3). Although this percentage is substantial, it is important to note that the confidence interval for this group was broad. This wide interval indicates that other factors or variables may play a role in determining the overall prognosis, and these should be carefully considered in future assessments.

Furthermore, the likelihood ratio (LR) results, which showed values of 1 and 2, suggested only a weak relationship between the examined factors and the likelihood of radioresistance. This weak association underscores the complexity of the issue and highlights the need for a more comprehensive evaluation that considers additional variables that may influence patient outcomes.

## 4. Discussion

Approximately 59.6% of individuals exhibited progressive diseases according to the RECIST 1.1 criteria. The threshold for LATS1 was 0.02765 ng/mg, with an area under the curve (AUC) of 32.7%. There was also a significant difference in the proportions of LATS1 concentrations above the cut-off between the radioresistant and radiosensitive groups (*p* < 0.001). The threshold for NF-κB was 192.42 pg/mg, with an AUC of 61%. There was a significant difference in NF-κB levels below the cut-off between the radioresistant and radiosensitive groups (*p* = 0.009).

These findings suggest that higher concentrations of LATS1 above the cut-off and NF-κB below the cut-off are linked to weaker responses to radiation therapy. The Hippo signaling pathway, referred to as the Salvador/Warts/Hippo (SWH) pathway, plays a crucial role in determining organ size in animals by inhibiting cell proliferation and apoptosis [25].

A previous study [26] found that LATS1 shows reduced expression in human cervical cancer and is associated with disease staging. In cervical cancer cell lines, LATS1 demonstrated an inhibitory effect on both the proliferation and invasion of cervical cancer cells, as predicted by the regulation of p27 and Matrix metalloproteinase-9 (MMP9). LATS1 was also found to activate the Hippo pathway through downregulation of Yes-associated protein (YAP) and connective tissue growth factor (CTGF). In contrast, another study [27] observed that LATS1/2 expression was elevated in oral squamous cell carcinoma. This increase was linked to the activation of SNAIL and TAZ (transcriptional co-activator with PDZ binding motif), which are involved in the epithelial–mesenchymal transition (EMT) and cell division. This study highlighted that LATS1/2 plays a crucial role in cancer stem cell renewal by regulating the Hippo pathway, EMT, and cell division.

A recent study has shown that the YAP oncogene enhances susceptibility to human papillomavirus (HPV) by increasing the expression of HPV receptor molecules and reducing the host’s innate immune response. Immunohistochemical analysis showed that YAP expression was significantly higher in cervical cancer tissues than in normal tissue and was associated with tumor stage, with higher levels observed in advanced cervical carcinoma. This, in turn, enhances the expression of transforming growth factor-β (TGF-β), activates epidermal growth factor receptor (EGFR), and inhibits LATS1 and LATS2, leading to cancer cell proliferation. Additionally, the HPV oncoproteins E6/E7 may help maintain YAP protein levels in cervical cancer cells, preventing its degradation [28].

In addition to LATS1, NF-κB was also found to play a significant role in radiation resistance. The results indicated that patients with progressive disease (PD) had NF-κB concentrations below the established cut-off, suggesting that decreased NF-κB levels correlate with poor radiation therapy responses. In a previous study, NF-κB expression was analyzed in 32 patients with advanced cervical cancer (97% stage IIB) who underwent radiation therapy followed by radical hysterectomy. Immunohistochemistry revealed NF-κB expression inside the cytoplasmic region or nucleus prior to radiation; however, it was not a reliable marker for predicting residual disease post-treatment. Notably, patients with residual disease after radiation exhibited decreased levels of p65 and p50 subunits, along with an increased expression of NF-κB-p50 compared to the original tumor, suggesting altered NF-κB signaling in radiation-resistant tumors [29].

Significant findings were observed in experiments conducted on cultured radioresistant cervical cancer cells (SiHa/RR cells). A study reported a notable increase in the expression of Aurora Kinase A (AURKA), accompanied by the activation of the HIF-1α, pAkt, and NF-κB signaling pathways. AURKA, a critical kinase involved in mitosis during the G2/M phase, plays a pivotal role in promoting radioresistance. This resistance mechanism is facilitated by a survival pathway that supports the translocation of NF-κB to the nucleus. The inhibition of AURKA using specific inhibitors led to increased radiation sensitivity and a decrease in the expression of the NF-κB subunits p50 and p65, which are downstream effectors of AURKA [30].

NF-κB can initiate autophagy by promoting the expression of specific genes and proteins such as the Beclin-1 complex, BCL-2-associated anthanogene-heat shock protein B8 (BAG3-HSPB8), autophagy-related 5 (ATG5), and light chain 3 (LC3). Autophagy plays a crucial role in modulating the NF-κB pathway by degrading IKK and IκBα complexes. IKK degradation can inhibit pro-tumoral NF-κB signaling, leading to tumor regression. Conversely, the degradation of IκBα can activate NF-κB, thereby promoting tumor growth. Various factors, including KEAP1, Ro52, Hsp90, SKP2, and SQSTM1/p62, influence the degradation targets within the NF-κB pathway [31]. Additionally, the concentration of NF-κB can be affected by reactive oxygen species (ROS) production, the thioredoxin peroxidase system [32], and other variables related to sample preparation and processing. Therefore, disease progression in patients with progressive disease (PD) is not solely determined by the NF-κB concentration.

**Figure 2:**
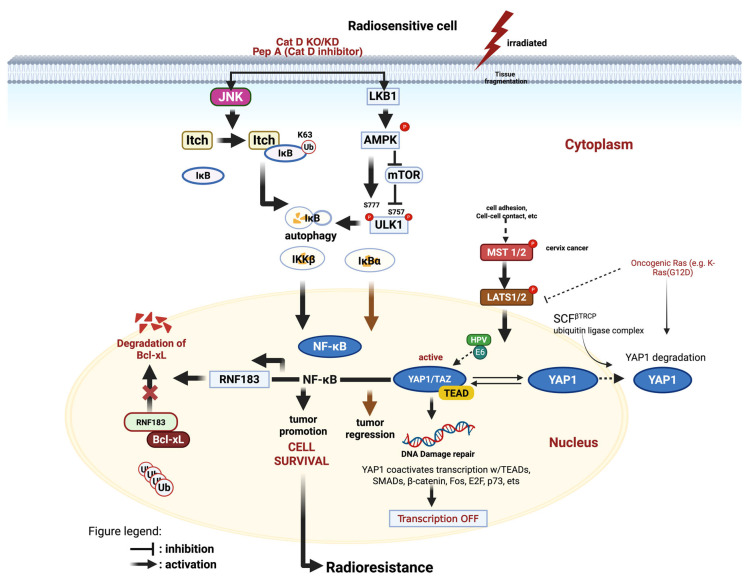
Radioresistance mechanisms in cervical cancer cells rely on LATS1 and NF-κB. Illustration created using Biorender.com.

The mechanisms underlying radioresistance in cervical cancer cells are visually summarized in Figure 2. The diagram highlights the involvement of LATS1/2 and NF-κB in radiation resistance pathways. When radiosensitive cells are irradiated, the liver kinase B1-AMP-activated protein kinase (LKB1-AMPK) pathway inhibits mTOR and activates unc-51-like kinase 1 (ULK1), as well as the Jun N-terminal kinase (JNK)-Itch pathway, resulting in increased autophagy and NF-κB activity [33,34,35]. In the nucleus, NF-κB promotes cell survival by upregulating ring finger protein 183 (RNF183), which mediates the degradation of the anti-apoptotic protein Bcl-xL, thereby facilitating tumor growth [33]. Conversely, LATS1/2 typically prevents YAP1 activity by inducing its degradation. However, in cases such as cervical cancer, oncogenic mutations like Ras (G12D) or the loss of MST1/2 function can disrupt this regulatory control, allowing YAP1 to remain active [36]. YAP1/TAZ then connects with the transcription factor TEAD (transcriptional enhancer activator domain) [37] in the nucleus, which initiates the expression of DNA repair genes, further promoting tumor cell survival and enhancing resistance to radiation [38,39,40,41]. Therefore, an imbalance in the activities of LATS1/2 and NF-κB contributes to the increased radioresistance observed in cervical cancer cells undergoing radiation therapy.

This study has several limitations. First, the analysis was conducted only prior to radiation treatment, with no assessment of post-irradiation variables. However, the primary aim of this study was to investigate predictive factors for radiation response rather than to examine changes in variable concentrations between pre- and post-treatment. Second, this study focused on locally advanced cervical cancer, which differs from other studies that have investigated the effects of radiotherapy as a definitive therapy in early-stage cancer. The decision to include patients with locally advanced stages was based on the fact that most cervical cancer patients in Indonesia are diagnosed at an advanced stage, which is associated with a poor prognosis. As such, research on predictive factors of therapeutic response in this population is crucial for improving patient management and extending survival.

Additionally, this study considered a limited number of variables, which may not fully capture the many factors influencing disease progression before and after treatment. Finally, the findings suggest that studies involving patients from routine clinical practice may produce results that differ from those based on earlier research due to variations in subject characteristics and research methodologies.

## 5. Conclusions

This study explored the roles of LATS1 and NF-κB as potential biomarkers for radioresistance in cervical cancer, specifically examining their relationship with radiation therapy response. The results indicate that higher concentrations of LATS1 above the threshold and lower concentrations of NF-κB below the cut-off are significantly associated with weaker responses to radiation therapy.

However, these findings diverge from some established theories and prior research. This discrepancy suggests that the role of LATS1 may not be universally consistent and could vary depending on the cancer type and context. Similarly, while NF-κB is commonly recognized as a key mediator of radioresistance, our study identified an inverse relationship between NF-κB levels and radiation response, with lower NF-κB concentrations correlating with poorer outcomes. These results challenge traditional perspectives on the function of NF-κB in cervical cancer, highlighting the need for a more nuanced understanding of its role in radioresistance.

These findings contribute to the growing body of evidence supporting personalized treatment strategies based on molecular biomarkers. Nonetheless, the observed discrepancies with existing theories underscore the necessity for further investigation into the mechanisms through which LATS1 and NF-κB influence radioresistance. Future research should incorporate a broader range of variables, including genetic, molecular, and environmental factors, as well as patient-reported outcomes and quality of life measures.

## Figures and Tables

**Table 1:** Characteristics of research subjects (*n* = 114).

Variable	Q1–Q4	*n* (%)
Age (years)	53 (43–79)	
Marital status		
No information		2 (1.7)
Not married		4 (3.5)
Married		87 (76.3)
Married > 1 time(s)		21 (18.4)
Length of marriage (years)	24 (18–43)	
Parity	3 (2–7)	
0		8 (7)
1		12 (10.5)
2		34 (29.8)
>2		60 (52.6)
Abortion	0 (0–2)	
0		86 (75.4)
1		21 (18.4)
2		7 (6.1)

**Table 2:** Analysis of LATS1 and NF-κB expression and radiation response in advanced stage cervical cancer patients.

Biomarkers	Radiosensitive (CR/PR)	Radioresistant (SD/PD)	Total	*p*-Value *
	Median (Q1–Q4)	Median (Q1–Q4)	Median (Q1–Q4)	
LATS1 (ng/mg)	8.6 × 10^−3^ (2.9 × 10^−3^–7.6 × 10^−2^)	4.9 × 10^−2^ (1.5 × 10^−2^–1.1 × 10^−1^)	3.2 × 10^−2^ (6.3 × 10^−3^–1 × 10^−1^)	0.001
NF-κB (pg/mg)	438.7 (71.2–736.3)	291.7 (10.3–423.5)	351 (23.1–622)	0.045

Q1–Q4: quartile 1–quartile 4; CR: complete response; PR: partial response; SD: stable disease; PD: progressive disease. * Mann–Whitney test, significant if *p* < 0.05.

**Table 3:** Prediction of radiation response based on LATS1 and NF-κB expression using post-test probability.

Biomarkers	*n*	Pre-Test Probability	Se	Sp	PPV	NPV	LR	Pre-Test Odds	Post-Test Odds	Post-Test Probability	95% CI
LATS1	114	59.6	66.2	65.2	73.8	56.6	1.7	1.48	2.51	71.5	62–79
NF-κB	114	59.6	61.8	63.0	71.2	52.7	1.5	1.48	2.23	69.0	60–77

Se: sensitivity; Sp: specificity; PPV: positive predictive value; NPV: negative predictive value; LR: likelihood ratio.

## Data Availability

The data that support the findings of this study are available from Open Science Framework (DOI: 10.17605/OSF.IO/5P2F6): Investigating the relationship between radiotherapy response and the LATS1 and NF-κB ratio as potential biomarkers in the inhibition of tumors in cervical cancer stages 2–3. This project includes the subsequent foundational data: Expression of LATS1 and NF-κB in response to radiation; ROC curves for LATS1 and NF-κB; Predicting the effects of radiation on the expression of LATS1 and NF-κB.
